# Recent Advances in Nanomaterials-Based Chemo-Photothermal Combination Therapy for Improving Cancer Treatment

**DOI:** 10.3389/fbioe.2019.00293

**Published:** 2019-10-22

**Authors:** Zuhong Li, Yangjun Chen, Ya Yang, Yan Yu, Yanhong Zhang, Danhua Zhu, Xiaopeng Yu, Xiaoxi Ouyang, Zhongyang Xie, Yalei Zhao, Lanjuan Li

**Affiliations:** ^1^State Key Laboratory for Diagnosis and Treatment of Infectious Diseases, Collaborative Innovation Centre for Diagnosis and Treatment of Infectious Diseases, The First Affiliated Hospital, Zhejiang University School of Medicine, Hangzhou, China; ^2^School of Ophthalmology & Optometry, Eye Hospital, Wenzhou Medical University, Wenzhou, China; ^3^Department of Gynecologic Oncology, Women's Hospital, Zhejiang University School of Medicine, Hangzhou, China

**Keywords:** cancer, nanomaterials, NIR responsive, chemo-photothermal therapy, synergistic effect

## Abstract

Conventional chemotherapy for cancer treatment is usually compromised by shortcomings such as insufficient therapeutic outcome and undesired side effects. The past decade has witnessed the rapid development of combination therapy by integrating chemotherapy with hyperthermia for enhanced therapeutic efficacy. Near-infrared (NIR) light-mediated photothermal therapy, which has advantages such as great capacity of heat ablation and minimally invasive manner, has emerged as a powerful approach for cancer treatment. A variety of nanomaterials absorbing NIR light to generate heat have been developed to simultaneously act as carriers for chemotherapeutic drugs, contributing as heat trigger for drug release and/or inducing hyperthermia for synergistic effects. This review aims to summarize the recent development of advanced nanomaterials in chemo-photothermal combination therapy, including metal-, carbon-based nanomaterials and particularly organic nanomaterials. The potential challenges and perspectives for the future development of nanomaterials-based chemo-photothermal therapy were also discussed.

## Introduction

Traditional chemotherapy, typically the main treatment for late stage cancer or adjunct method for surgery in early stage cancer, usually suffers severe systemic toxicity due to the unspecific cytotoxicity of chemotherapeutic drugs for both cancerous and normal cells (Cobley et al., 2010; Mahmoudi et al., 2011; DeSantis et al., 2014). As a result, the outcome of chemotherapy is often limited by safe dosage, generating insufficient drug concentration in tumor site. Moreover, drug resistance is possibly developed to further hamper the overall efficacy during the treatment course (Holohan et al., 2013). Thus, it is urgent to improve specific delivery to reduce side effects, and optimize the therapeutic efficacy at a lower tolerance dose.

Hyperthermia as cancer therapy refers to the treatment of cancer through heating and has been used in various forms since the original study pioneered by Coley in the end of nineteenth century (Mallory et al., 2016). Although it can be used alone, hyperthermia is most often used in combination with other therapeutic modalities including chemotherapy and radiation therapy. Hyperthermia typically falls under three categories: local hyperthermia, regional hyperthermia, and whole-body hyperthermia. Clinical application of heat can be induced by radiofrequency, microwave, ultrasound, or perfusion methods (Falk and Issels, 2001). While these methods heat tissues efficiently, they also cause either a risk of systemic toxicity from whole-body hyperthermia exposure, require invasive surgery or probe, or may damage normal tissues due to non-targeted heating in local region (Wust et al., 2002). Thus, photothermal therapy has been proposed as a promising modality for hyperthermia treatment. Compared with other methods, light is an ideal external stimulus as it is easily regulated, focused, and remotely controlled. The ease of control and focus enables better targeted treatments and leads to less damage in healthy tissues.

NIR photothermal therapy as an emerging strategy, utilizing NIR laser-generated heat to conduct cancer treatment, has gained increasing attentions (Peng et al., 2011; Wu et al., 2013; Yue et al., 2013). In NIR window, NIR light is minimally absorbed by endogenous absorbers in tissues, which offers deeper tissue penetration *in vivo* (Weissleder, 2001; Weissleder and Ntziachristos, 2003). Besides the heat ablation for direct cell killing in tumor, NIR light-induced mild hyperthermia can increase vascular permeability in tumor tissues with newly formed immature blood vessels, which brings specific drug accumulation and enhanced cytotoxicity (Hauck et al., 2008; Park et al., 2009). Various kinds of nano-structured materials, including both organic and inorganic nanomaterials, have been designed and applied for photothermal therapy as shown in several excellent reviews (Jung et al., 2018; Khafaji et al., 2019; Vines et al., 2019). However, due to the non-uniform heat distribution and restricted laser power to avoid normal tissue damage, the photothermal therapy alone is unlikely to eradicate tumor completely (Wang H. et al., 2013; Luo et al., 2017).

To address these issues, nanomaterials-based combination of chemotherapy and hyperthermia has exhibited the effectiveness in optimizing the efficacy for cancer treatment (You et al., 2012; Zheng et al., 2013; Wang L. M. et al., 2014). It is well-known that nanomedicines can preferentially accumulate in tumor site through passive targeting via enhanced permeability and retention (EPR) effect, or active targeting via surface-conjugated molecules (Jain and Stylianopoulos, 2010; Kratz and Warnecke, 2012). Their unique physicochemical properties also offer different pharmacokinetics and *in vivo* distribution for loaded chemotherapeutic agents (Ernsting et al., 2013). In another hand, nanomaterials-mediated NIR photothermal therapy is finely localized inside the tumor region, and the hyperthermia is tunable simply by controlling the timing and intensity of the extrinsic energy source (Kim et al., 2016). It has been widely accepted that combined chemo-photothermal therapy based on nanomaterials exhibits remarkable advantages over single cancer treatment. Generally, co-delivery of cytotoxic drugs and hyperthermia can simultaneously exert two benefits to improve cancer treatments, and combined chemo-photothermal therapy usually generates synergistic effect. Photothermal ablation coupled with targeted drug delivery can synergistically enhance therapeutic index via different manner: (i) elevating cell membrane permeability; (ii) augmenting drug cytotoxicity (Hahn et al., 1975; Overgaard, 1976); (iii) triggering drug release at target region. This can be especially significant in treating cancers with multidrug resistance (MDR) (Wang L. M. et al., 2014). So far, there have been several related reviews published, reporting either organic or inorganic nanomaterials for chemo-photothermal combination therapy (Zhang et al., 2013; Zhang A. et al., 2018; Khafaji et al., 2019). Considering the rapid development of this research area, we believe it is highly desirable and important to systematically summarize the recent advances in combined chemo-photothermal therapy based on both organic and inorganic nanomaterials.

Herein, we will review the recent efforts to design and construct nanomaterials for cancer chemo-photothermal therapy. This topic will be presented based on the properties and classifications of nanomaterials applied as photothermal agents and nanocarriers. Upon briefly elaborating new progress in metal and carbon nanomaterials mediated chemo-photothermal therapy, organic nanomaterials-based combination therapy was discussed in particular. Material design and formulations for integrated drug delivery and NIR-responsive hyperthermia are highlighted on the background of their potential capacity in optimizing efficacy of cancer treatment.

## Metal Nanomaterials-Based Chemo-Photothermal Therapy

### Gold Nanoparticles

**Figure 1:**
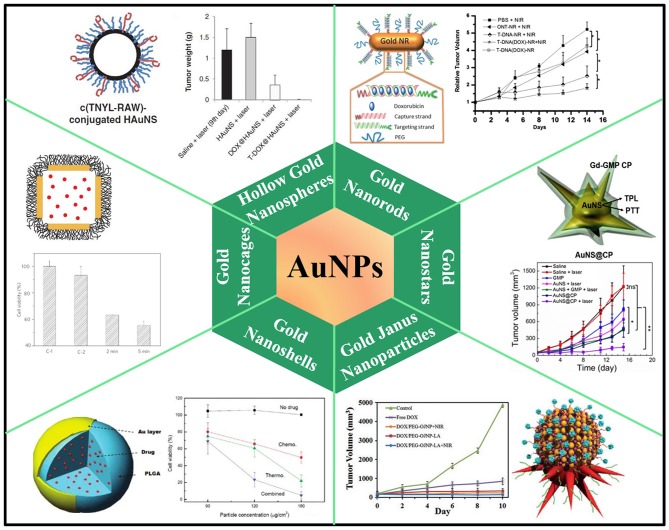
AuNPs-based nanoplatforms for NIR light-responsive chemo-photothermal therapy. Hollow gold nanospheres, reproduced with permission from You et al. (2012); Gold nanorods, reproduced with permission from Xiao et al. (2012); Gold nanocages, reproduced with permission from Yavuz et al. (2009); Gold nanstars, reproduced with permission from Li M. et al. (2016); Gold nanoshells, reproduced with permission from Park et al. (2009); Gold Janus nanoparticles, reproduced with permission from Zhang L. et al. (2016).

As is well-known, gold nanoparticles (AuNPs) have been widely investigated in biomedical fields due to their unique size- and shape-dependent optical and photothermal properties, originating from localized surface plasmon resonance (LSPR) where collective oscillation of electrons occurs on the surface of AuNPs after light absorption at a certain frequency (Cobley et al., 2011; Dreaden et al., 2012; Saha et al., 2012). Following excitation of LSPR by NIR laser, the attenuation of resonance energy can occur through radiative and non-radiative relaxation, generating localized heat to surrounding medium. The heat converted from absorbed NIR light can be used to perform hyperthermia or trigger drug release in delivery systems (Hu et al., 2006; Dykman and Khlebtsov, 2012; Llevot and Astruc, 2012). AuNPs also exhibit chemical inertness and good biocompatibility in biological tissues (Khlebtsov and Dykman, 2011). All these properties make AuNPs a promising candidate for effective chemo-photothermal combination therapy (Figure 1). The synthesis of AuNPs with controlled size and morphology has obtained various nanostructures such as gold nanorods (Xiao et al., 2012; Ren et al., 2013; Shen et al., 2013; Manivasagan et al., 2019), gold nanoshells (Lee et al., 2010; Liu et al., 2011), hollow gold nanospheres (You et al., 2010, 2012), gold nanocages (Yavuz et al., 2009; Shi et al., 2012; Feng et al., 2019), and gold nanostar (Li M. et al., 2016; Zhang L. et al., 2016), whose LSPR absorption features can be finely tuned to NIR region. However, weak interactions between anticancer drugs and naked Au surface make them hardly attach to AuNPs for *in vitro* or *in vivo* co-delivery application. Thus, extra outer/inner layer for molecule absorption or pore-blocking strategy have been widely developed to load drug molecules for chemo-photothermal therapy. For example, thermo-sensitive amphiphilic block polymer, lipoic acid conjugated poly(ethylene glycol)-*b*-poly(ε-caprolactone), was employed to coat AuNRs surface for DOX loading via hydrophobic interactions. Light-triggered drug release after NIR irradiation was achieved due to the phase transition of poly(ε-caprolactone) on the AuNRs surface (Zhong et al., 2013). Mesoporous silica has also been widely explored to decorate AuNPs for drug delivery. High surface area, tunable pore size, and good biocompatibility make them suitable to augment drug loading capacity. For instance, mesoporous silica-coated AuNRs were developed to encapsulate DOX for chemo-photothermal therapy, exhibiting light-controlled drug release under low-intensity NIR laser irradiation (Zhang et al., 2012). DNA molecules can also serve as capping agents on the surface of AuNPs to realize drug encapsulation and release. DNA duplex strands, consisting of sequential CG base pairs, provide DOX loading sites on the AuNRs surface. Photothermal effects trigger de-hybridization of double-strand DNA by raising temperature higher than melting temperature to release DOX under light-controlled mode (Xiao et al., 2012). Hollow gold nanospheres and gold nanocages themselves could be employed as nano-carriers to load drugs into both outer and inner space. A well-known AuNC-based system was fabricated by coating AuNC surface with thermo-sensitive polymer [poly-(N-isopropylacrylamide), pNIPAAm] as pore blockers. Following temperature rise above a certain threshold, pNIPAAm layer collapsed and the pores on nanocages were exposed for interior drug release (Yavuz et al., 2009). The state of the art of AuNPs-based chemo-photothermal therapy has been wonderfully summarized in some review articles (Wang H. et al., 2013; Zhang et al., 2013; Ai et al., 2016; Kim et al., 2016).

Despite the tremendous attentions and encouraging results on AuNPs, some important issues should be addressed before further applications. On the one hand, AuNPs are subject to deformation upon high-power laser irradiation, leading to loss of LSPR absorption in the NIR region (Opletal et al., 2011; Young et al., 2012). On the other hand, the *in vivo* long-term toxicity and clearance pathways of AuNPs are still uncertain and require further study. To date, some studies have unveiled potential factors that have effect on AuNPs cytotoxicity. It is believed that surface charge and particle size are likely to be the most influential factors. It was reported that half the dose of positively charged 5 nm AuNPs were excreted after 5 days, while only about 10% of the dose of negatively or neutrally charged 5 nm particles were excreted (Balogh et al., 2007). Most of AuNPs accumulations occur within liver and spleen after intravenous injection of PEG-coated AuNRs (Glenn et al., 2010). Moreover, chronic inflammation was observed in tissues around these AuNPs, despite the unclear long-term consequence under this type of chronic inflammation. *In vivo* observation of AuNPs only take place up to 6 months in animal models, leaving unanswered questions about the potential influence on health over long time course.

### Palladium Nanosheets and Copper Chalcogenides Nanocrystals

**Figure 2:**
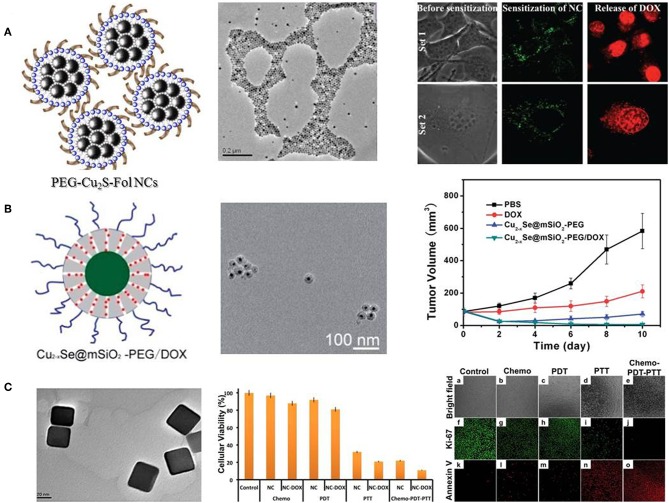
Different copper chalcogenides nanocrystals mediated chemo-photothermal therapy. **(A)** Schematic illustration and TEM image of PEG-Fol-Cu_2_S nanoparticles, and photoexcitation mediated drug release. Reproduced with permission from Poulose et al. (2015). **(B)** Schematic illustration and TEM image of Cu_2−x_Se@mSiO_2_-PEG nanoparticles, and synergistic effects on cell viability and tumor volume. Reproduced with permission from Liu et al. (2014). **(C)** TEM image of PEG-Cu_2−x_Te nanocubes and effects of chemo-photothermal treatment on cell viability and cellular apoptosis. Reproduced with permission from Poulose et al. (2016).

Another noble metal-based nanostructure, Pd nanosheets, has also been developed to conduct chemo-photothermal therapy. Pd nanosheets exhibit tunable LSPR peaks in the NIR region, as well as photothermal stability, thermal transformation efficiency and biocompatibility (Huang et al., 2011). To facilitate the hyperthermia-assisted chemotherapy, Pd nanosheets were deposited onto hollow mesoporous silica particles or coated with a mesoporous silica layer to achieve drug release under NIR irradiation and low pH (Fang et al., 2012a,b). Besides noble metal-based nanomaterials, copper chalcogenides nanocrystals have been supposed to be a promising photothermal agent in biomedical applications. Being p-type semiconductors, copper chalcogenides nanocrystals provide composition-dependent LSPR in NIR region and high photothermal conversion efficiency (Hessel et al., 2011; Lie et al., 2014). Kumar et al. decorated Cu_2_S nanocrystals with polyethylene glycol (PEG) and folate (PEG-Fol-Cu_2_S) to physically absorb doxorubicin (DOX) for multimodal therapeutics against brain cancer cells (Poulose et al., 2015). The folate targeted photothermal ablation in synergism with photo-responsive DOX release proved to be a rapid precision guided cancer-killing module (Figure 2A). Hu et al. reported a low-toxic di-functional nanoplatform based on Cu_2−x_Se@mSiO_2_-PEG core-shell nanoparticles for cancer treatment (Liu et al., 2014). DOX was loaded into mesoporous silica shell, and the release of DOX can be triggered by pH and NIR laser, resulting in a synergistic effect in anti-tumor therapy. The chemo-photothermal therapy driven by NIR radiation with safe power density significantly improved the therapeutic efficacy (Figure 2B). In another work, DOX loaded PEG-Cu_2−x_Te nanocubes were developed for treatment of hypermethylated breast cancer cells (Poulose et al., 2016). PEG-Cu_2−x_Te/DOX nanocubes conducted highly effective chemo-photothermal-photodynamic therapy to overcome hypermethylated cancer cells resisting to chemotherapeutic drugs (Figure 2C).

### Two-Dimensional Transition Metal Dichalcogenides

In recent years, a class of two-dimensional transition metal dichalcogenides (2D TMDCs) has attracted tremendous attention. 2D TMDCs are typically made up of a layer of transition metal atomssandwiched between two layers of chalcogen atoms. Their generalized formula is MX_2_, where M is a transition metal of groups 4-10 (Mo, W, Ti, Ta, Zr, V, Nb, etc.) and X is a chalcogen (Chhowalla et al., 2013). Single-layered 2D TMDCs exhibit superior properties, such as strong NIR absorbance, high photothermal conversion efficiency as well as good photothermal stability, offering the possibility to be excellent photothermal agents (Wang C. et al., 2016; Zhu et al., 2017). Moreover, the ultra-high surface area of 2D TMDCs endows themselves with efficient cargo loading ability as drug carriers for chemotherapy.

**Figure 3:**
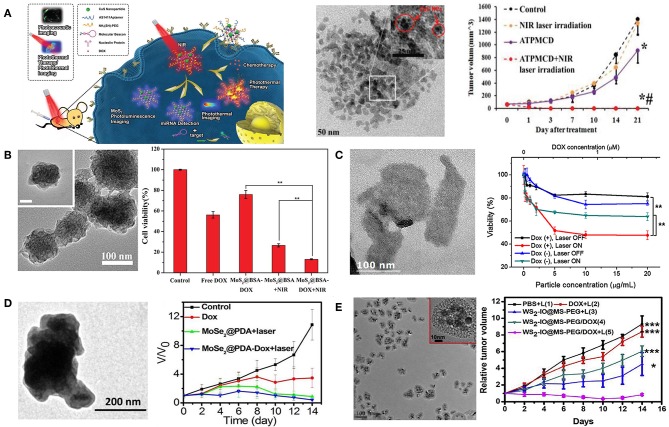
2D TMDCs for combined chemo-photothermal therapy. **(A)** Schematic illustration of DOX loaded ATPMC for NIR-laser irradiation-induced chemotherapy. TEM image of MoS_2_/Cu_1.8_S nanosheets and tumor volume change with different treatments (*P < 0.01 compared with control group; ^#^*P* < 0.01 compared with ATPMCD group). Reproduced with permission from Meng et al. (2017). **(B)** FESEM image of MoS_2_@BSA nanoparticles and combined therapeutic efficacy of MoS_2_@BSA-DOX in 4T1 cells (**P < 0.01 compared with MoS_2_@BSA-DOX + NIR group). Reproduced with permission from Chen L. et al. (2016). **(C)** TEM image of MoS_2_@SiO_2_-NH_2_ nanoplates and NIR-induced cytotoxicity of DOX-MoS_2_@SiO_2_-PEG against HepG2 cells (**P < 0.01). Reproduced with permission from Lee et al. (2016). **(D)** TEM image of MoSe_2_@PDA nanocomposites and tumor growth curves of different treatments. Reproduced with permission from Wang C. et al. (2016). **(E)** TEM image of WS_2_-IO@MS-PEG and growth of 4T1 tumors in different groups of mice after various treatments (***P < 0.001, *P < 0.05). Reproduced with permission from Yang G. B. et al. (2015).

Meng et al. prepared aptamer conjugated PEG-MoS_2_/Cu_1.8_S nanosheets (ATPMC) as multifunctional platforms for chemo-photothermal therapy (Meng et al., 2017). ATPMC nanoplatform possessed superb photothermal conversion efficiency due to the interactions of MoS_2_/Cu_1.8_S nanocomposites. DOX loaded ATPMC displayed NIR laser-induced programmed chemotherapy and advanced photothermal therapy, and the targeted chemo-photothermal therapy presented excellent antitumor efficiency (Figure 3A). In another work, flower-like MoS_2_ nanoparticles coated with bovine serum albumin (BSA) were successfully fabricated to load DOX for cancer treatment (Chen L. et al., 2016). Fabricated MoS_2_@BSA-DOX exhibited high photothermal conversion efficiency as well as intelligent drug release. Combination of DOX release and photothermal treatment displayed better therapeutic efficacy than single photothermal therapy or chemotherapy (Figure 3B). Kim et al. reported a photothermally controllable DOX-MoS_2_@SiO_2_-PEG nanoplate as functional drug delivery carrier (Lee et al., 2016). DOX release was facilitated by external NIR laser irradiation under acidic pH. Enhanced anticancer effect of DOX-MoS_2_@SiO_2_-PEG was achieved by the combination of heat damage and enhanced DOX release as well as endosomal escape (Figure 3C).

Beside MoS_2_ nanosheetes, other 2D TMDCs have also been studied for the application of chemo-photothermal therapy (Wang Y. et al., 2019). Jiang et al. successfully prepared MoSe_2_@PDA-DOX to be applicable for multi-modal chemo-photothermal therapy (Wang C. et al., 2016). MoSe_2_@PDA-DOX nanocomposites showed high loading efficiency and NIR-responsive DOX release. Synergistic therapy significantly inhibited cancer cell viability, and suppressed *in vivo* tumor growth (Figure 3D). In addition, Liu et al. fabricated WS_2_nanosheets with iron oxide (IO) nanoparticles on surface, subsequently coated with mesoporous silica shell and PEG (Yang G. B. et al., 2015). The obtained WS_2_-IO@MS-PEG nanocomposites exhibited high NIR light and X-ray absorbance as well as NIR-responsive DOX release. Chemo-photothermal therapy based on WS_2_-IO@MS-PEG/DOX achieved a remarkable synergistic effect superior to the respective mono-therapies (Figure 3E).

As other metal nanomaterials, biosafety is one of the most concerning issues for 2D TMDCs in biomedical applications, while the knowledge about toxicity for 2D TMDCs is very limited. Till now, only a few 2D TMDCs have been tested for their *in vitro* and *in vivo* cytotoxicity, and further studies should be systematically carried out to examine their acute and long-term cytotoxicity.

## Carbon-Based Chemo-Photothermal Therapy

Over the past few decades, a range of carbon nanoallotropes with surprising properties and diverse potential applications have been discovered, such as carbon nanotubes (Iijima, 1991), graphene (Novoselov et al., 2004), carbon dots (Xu et al., 2004), fullerene (Kroto et al., 1985), and nanodiamonds (Niwase et al., 1995). The majority of the interest in carbon nanomaterials for biomedical applications relates to bio-imaging and cancer treatment because of their extraordinary photon-to-thermal conversion efficiency and ultrahigh surface area (Lim et al., 2014), as well as the ability to integrate different biomolecules and drugs on a nanoscale platform, generating advanced hybrid delivery systems.

### Graphene/Graphene Oxide

Graphene is a two-dimension shaped carbon nanoallotrope, in which carbon atoms are arranged in a single atom thick sheet packed into a honeycomb lattice (Novoselov et al., 2004). The unique feature of graphene provide tremendous tunable surface with great mechanical Young's modulus, fracture strength, electrical, thermal and optical properties (Yang et al., 2010; Feng and Liu, 2011). Graphene oxide (GO) is a highly oxidized form of graphene that is comprised of single atom carbon sheet with carboxylate groups on the border areas, and hydroxyl, epoxide groups on the basal surface. Carboxylate groups provide negatively charged surface and colloidal stability, whereas basal planes offer π-π interaction for the absorption of drug molecules. Like other nanomaterials-based photothermal agents, PEGylated GO and reduced GO exhibit high NIR absorbance and capacity in photothermal treatment (Robinson et al., 2011; Yang et al., 2012a; Zaharie-Butucel et al., 2019). Meanwhile, due to the ultrahigh surface area and delocalized π electron, many anticancer agents such as camptothecin (CPT), DOX, and 7-ethyl-10-hydroxycamptothecin (SN38) have been successfully loaded onto the GO surface (Li et al., 2006; Zhang et al., 2010; Bao et al., 2011; Pan et al., 2011). The works concerning on nano-graphene based chemo-photothermal therapy have been well-reviewed (Yang et al., 2013; Orecchioni et al., 2015; Rahman et al., 2015; Yang K. et al., 2015). For biomedical application of nano-graphene, one of the critical issues is still the potential long-term toxicity in biological systems (Kiew et al., 2016). It is urgent to figure out that whether and how nano-graphene would be gradually degraded in living body, which is essentially unclear at present and needs a lot more efforts in future studies.

### Carbon Dots and Mesoporous Carbon Nanoparticles

**Figure 4:**
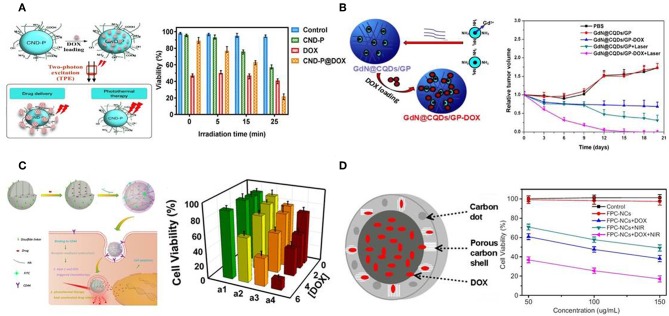
C-dots- and MCNs-based chemo-photothermal therapy. **(A)** Schematic illustration of CND-P and cell viability with different treatments. Reproduced with permission from Ardekani et al. (2017). **(B)** Schematic illustration of GdN@CQDs/GP and relative tumor volume of tumor-bearing mice after various treatments. Reproduced with permission from Zhang et al. (2017a). **(C)** Schematic illustration of MCNs, and cytotoxicity assays of MDA-MB-231 cells under different treatments. Reproduced with permission from Zhou et al. (2015). **(D)** Schematic illustration of FPC-NCs and *in vitro* cytotoxicity after various treatments. Reproduced with permission from Wang H. et al. (2015).

Carbon dots (C-dots) are quasi-spherical carbon nanoparticles with diameters of 2–10 nm, and consist of oxygen, nitrogen elements and other doped heteroatoms (Baker and Baker, 2010). Due to their high quantum yield, superior chemical and photostability, low cytotoxicity and low cost, C-dots are generally regarded as a promising candidate in cancer therapeutic applications (Zhao et al., 2015; Zheng et al., 2015; Wang and Qiu, 2016). Gomes et al. prepared PEG_2000_ passivated nitrogen-doped C-dots (CND-P) to remotely initiate the delivery of DOX in 3D cultured MCF-7 cells (Ardekani et al., 2017). CND-P possessed high drug loading capacity with the ability to release DOX under two-photon excitation. CND-P/DOX mediated chemo-photothermal treatment was superior to single treatment of CND-P or DOX in killing efficiency (Figure 4A). Moreover, magnetofluorescent C-dots were also reported as chemo-photothermal therapeutic agents (Zhang et al., 2017a,b). For example, Zhou et al. prepared Gd doped magnetofluorescent C-dots (GdN@CQDs) as drug carriers, followed with cross-linking by genipin to form multifunctional delivery system (GdN@CQDs/GP-DOX) with pH- and NIR-triggered drug release (Zhang et al., 2017a). GdN@CQDs/GP demonstrated strong NIR absorption and high photothermal conversion efficiency. Upon laser irradiation, GdN@CQDs/GP-DOX has the ability to achieve synergistic therapeutic effect (Figure 4B).

Recently, mesoporous carbon nanoparticles (MCNs) have received considerable attention in the family of carbon-nanomaterials (Fang et al., 2010). This growing interest stems from their intrinsic properties, such as high surface areas, large pore volumes, and well-defined surface properties, offering significant advantages as drug carrier on the basis of higher loading capacity and biologic inertness without cytotoxicity (Karavasili et al., 2013). Furthermore, MCNs could also be used as NIR-absorbing nanomaterials with high photothermal conversion efficacy (Dong et al., 2016; Wang X. et al., 2019). Qu et al. developed hyaluronic acid surface-modified MCNs (MCNs-HA) as nanocarriers for effective dual-triggered synergistic cancer therapy (Zhou et al., 2015). This system was sensitive to both intracellular hyaluronidase-1 and GSH level to release loaded DOX. In combination with photothermal therapy, DOX-MCNs-HA showed effective therapeutic efficiency toward target cells (Figure 4C). In another study, hollow porous carbon nanoparticles with C-dots embedded in carbon shell (FPC-NCs) were fabricated (Wang H. et al., 2015). Such prepared FPC-NCs demonstrated great potential to combine multiple functions for simultaneous two-photon cell imaging, responsive drug delivery, and photothermal therapy. DOX-loaded FPC-NCs manifested NIR-responsive drug release and combined chemo-photothermal therapy (Figure 4D).

## Organic Nanomaterials-Based Chemo-Photothermal Therapy

In spite of the excellent photothermal conversion efficiency, the poor biodegradability and potential long-term toxicity of inorganic nanoparticles are still major obstacles for their clinical applications in the future (Jung et al., 2018). In this regard, organic nanoparticles usually exhibit optimized biodegradability and biocompatibility as an alternative approach for combined chemo-photothermal therapy in cancer treatment (Shi et al., 2017; Pierini et al., 2018). Additionally, organic nanoparticles have other attractive advantages such as facile preparation under mild conditions, desirable photothermal features based on easily tuning molecular structures, and satisfactory drug loading efficiency (Yue et al., 2017). In this section, we will mainly talk about small molecular NIR dyes, conjugated polymers and melanin-like polydopamine based organic nano-systems and their applications in synergistic chemo-photothermal therapy.

### Small Molecular NIR Dyes

Over the past few years, small molecular NIR dyes have attracted lots of attention mainly due to their excellent performance in fluorescent imaging and the ease of tuning photothermal features through elaborate chemical design and synthesis. In principle, these organic dyes with strong NIR absorbance can serve as photothermal agents as well (Song et al., 2015). Therefore, nano-systems containing NIR dyes can be easily designed as a nano-platform for multifunctional theranostics. To date, the well-studied NIR dyes include porphyrin, cyanine derivatives, borondipyrromethane dyes, diketopyrrolopyrrole derivatives and so on (Cai et al., 2018). Direct use of NIR dyes is mainly hindered by the rapid blood clearance following with undesired aggregation caused by the poor aqueous solubility/stability and non-specific protein adsorption (Chen Y. J. et al., 2016). From this point of view, NIR dyes are similar to many anticancer drugs which usually need to be administrated in the form of nano-formulations to improve therapeutic efficacy. Therefore, rational design of nano-systems with drugs/NIR dyes either encapsulated or conjugated is of great significance to achieve favorable synergistic outcomes of chemo-photothermal therapy.

The simplest way to combine NIR dyes and chemotherapeutic drugs is co-encapsulation in one nanoparticle by physical interactions. Cai et al. prepared poly(lactic-co-glycolic acid) (PLGA)-lecithin-PEG nanoparticles (DINPs) containing both DOX and indocyanine green (ICG) by a single-step sonication method (Zheng et al., 2013). ICG is the quintessential NIR dye and has been approved by US Food and Drug Administration (FDA) for several clinical applications (Sheng et al., 2013; Porcu et al., 2016). The stability of ICG was significantly improved by encapsulation in DINPs, thus generating higher localized temperature than free ICG under NIR laser irradiation and facilitating DOX release and cellular uptake. Encouragingly, combined chemo-photothermal therapy realized successful suppression of MCF-7 and DOX-resistant MCF-7/ADR tumor growth as well as prevention of tumor recurrence *in vivo*. Similarly, many other attempts have been made to co-encapsulate various NIR dyes and drugs in different kinds of nano-carriers like liposomes (Li et al., 2015; Feng et al., 2016; Yan et al., 2016; Gao et al., 2018; Mu et al., 2019), polymeric nanoparticles (Su et al., 2015; Zhu et al., 2015; Chen Y. et al., 2017; Wang et al., 2017; Deng et al., 2018; He H. Z. et al., 2018; Yao et al., 2018; Tan et al., 2019; Zhang et al., 2019), protein nanoparticles (Chen et al., 2015; Lin and Shieh, 2018; Gao et al., 2019; Pei et al., 2019), and cell membranes (Sun et al., 2015; Li X. et al., 2018; Wan et al., 2018; Zhang N. et al., 2018; Ye et al., 2019). For example, Liu et al. developed “Abraxane-like” nanodrug through self-assembly of human serum albumin (HSA), paclitaxel (PTX), and ICG in a simple mixing manner (Chen et al., 2015). The nanodrug remarkably improved solubility of both PTX and ICG, achieving prolonged blood circulation time and high tumor accumulation. The combined chemo-photothermal therapy completely destructed subcutaneous tumors and exhibited great therapeutic benefit in treating lung metastasis. The excellent therapeutic effect and FDA-approved components endow this “Abraxane-like” nanodrug with great potential for clinical use in the future.

**Figure 5:**
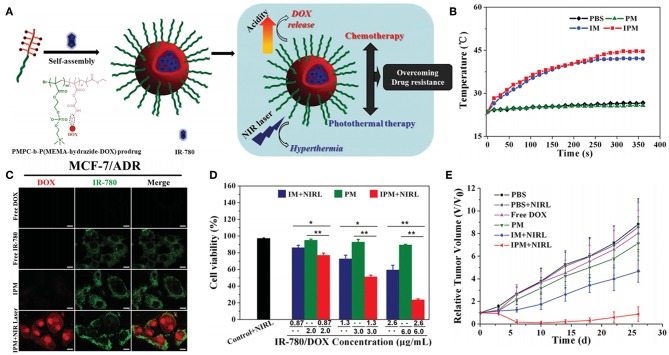
Polymeric prodrug micelles as nano-carriers for NIR dye. **(A)** Schematic representation of IR-780 loaded polymeric prodrug micelle for chemo-photothermal therapy to overcome drug resistance. **(B)** Temperature increments of IR-780 loaded micelles upon 808 nm NIR laser irradiation. **(C)** Subcellular localization of free DOX, free IR-780, IPM, and IPM plus NIR laser irradiation after coincubation with MCF-7/ADR cells. **(D)** Quantitative evaluation of cell viability in MCF-7/ADR cells after different treatments (*P < 0.05, **P < 0.01). **(E)** MCF-7/ADR tumor growth curves subjected to different treatments. Reproduced with permission from Li Z. H. et al. (2016).

The simple processing method of physically co-encapsulated nano-formulations is usually preferred for clinical translation. However, undesirable premature leakage of drugs and NIR dyes during administration can happen and lead to insufficient tumor accumulation and potential adverse effects as well. Covalent conjugation of drugs and/or NIR dyes to nano-carriers is believed to potently address this issue. Polymeric prodrugs with therapeutic molecules reversibly linked to polymer chains have already been extensively studied for their longer blood circulation time, higher tumor accumulation and better therapeutic effect but lower systemic toxicity (Duncan, 2006; Larson and Ghandehari, 2012; Delplace et al., 2014). Therefore, polymeric prodrug assemblies can serve as a good platform for co-loading of NIR dyes to perform combined chemo-photothermal therapy. Ji et al. prepared IR-780 loaded polymeric prodrug micelles (IPM) for overcoming multidrug resistance by combination of chemo-photothermal therapy (Figure 5; Li Z. H. et al., 2016). Zwitterionic polymer with DOX conjugated by acid-cleavable hydrazone bonds can self-assemble into polymeric prodrug micelles, simultaneously encapsulating lipophilic IR-780 dye in the hydrophobic micellar core. The prodrug micelles kept remarkable stability at pH 7.4 while exhibited accelerated DOX release at pH 5.0 which mimics the acidic endosome/lysosome environment. Interestingly, IPM combined with NIR laser irradiation can dramatically increase DOX accumulation in cytoplasm of MCF-7/ADR cells, which should be attributed to hyperthermia-induced enhanced cytoplasm permeability. Significant suppression of MCF-7/ADR tumor growth can be achieved by localized NIR laser irradiation post-intravenous injection of IPM. Recently, several similar strategies have been reported by encapsulating various NIR dyes in different kinds of polymeric prodrug nanoparticles (Zhang Y. Y. et al., 2016a,b, 2018; Chen et al., 2018; Wang W. H. et al., 2018). For example, Hu and Xing et al. prepared smart nanoplatforms by loading ICG in enzyme-responsive cisplatin polyprodrug amphiphiles for cascade photo-chemotherapy (Wang W. H. et al., 2018). Zhao et al. utilized host-guest supramolecular chemistry to fabricate reduction-sensitive CPT prodrug nanoparticles, which can further incorporate IR825 dye to achieve simultaneous chemo-photothermal therapy (Zhang Y. Y. et al., 2018).

Nano-systems with both drugs and NIR dyes covalently conjugated, however, have rarely been reported. The multiple steps that are usually necessary for conjugating two or more molecules in one single system may be the major obstacle. One of the very few examples is P-DOX/P-cypate hybrid micelles, which were composed of enzyme-responsive DOX polymeric prodrugsandcypate-linked polymers (Yu et al., 2015). Hyperthermia effect of the hydride micelles upon NIR irradiation significantly enhanced tumor penetration and cytosol release of DOX, further inducing high therapeutic efficacy in combating DOX resistance in MCF-7/ADR breast cancer.

Considering the tremendous benefits of NIR dyes-based nano-systems whereas few applications so far, further efforts are needed to simplify the synthesis procedures and precisely control the loading ratios of chemical drugs and NIR dyes, thus enabling ease of availability and optimizing the synergistic effect of chemo-photothermal therapy.

### Conjugated Polymers

Conjugated polymers are well-known for their fantastic photoelectric properties due to the existence of large π-conjugated backbones. For a long time in the past, conjugated polymers were extensively studied in the areas like organic semiconductors and solar cells, but not biomedicine. It was not until the year of 2011–2012 that two kinds of very commonly used conjugated polymers, polyaniline (PANI) and polypyrrole (PPy), were successfully developed as photothermal agents for the first time (Yang et al., 2011, 2012b). Later on, lots of semiconducting polymers were further explored for fluorescent imaging and phototherapy (Li J. C. et al., 2018; Zhu et al., 2018). Benefiting from the unique chemical structures, conjugated polymers hold the advantages of excellent photostability and high photothermal conversion efficiency. Several excellent reviews have summarized the applications of conjugated polymers in fluorescent imaging and phototherapy (Xu et al., 2014; Qian et al., 2017; Li J. C. et al., 2018; Sun et al., 2018). Conjugated polymers are generally hydrophobic organic molecules, therefore, nano-stabilizing methods like encapsulation in amphiphiles or covalent grafting of hydrophilic polymers are usually necessary prior to their use *in vitro* and *in vivo*. By further incorporation of chemical drugs, conjugated polymers based nanomaterials can be utilized for combined chemo-photothermal therapy.

PANI nanoparticles were firstly reported to serve as a photothermal agent for *in vitro* treatment of epithelial cancer in 2011 (Yang et al., 2011). Li et al. further prepared F127 stabilized PANI nanoparticles for *in vivo* tumor ablation with no tumor regrowth observed (Zhou et al., 2013). Chemotherapeutic drugs were encapsulated within PANI nanoparticles to perform the combination of chemo- and photothermal therapy. For example, Kim and Yong et al. prepared multifunctional hybrid polymeric nanoparticles (denoted as LT-MTX/PANI NPs) by incorporation of methotrexate (MTX) and PANI together and further conjugation with Lanreotide (LT) for cancer targeting (Nguyen et al., 2018). The hybrid NPs showed burst release of MTX upon NIR light irradiation due to heat generation by PANI. Enhanced cell apoptosis *in vitro* as well as improved tumor suppression *in vivo* were observed by treatment of LT-MTX/PANI NPs with NIR light irradiation.

**Figure 6:**
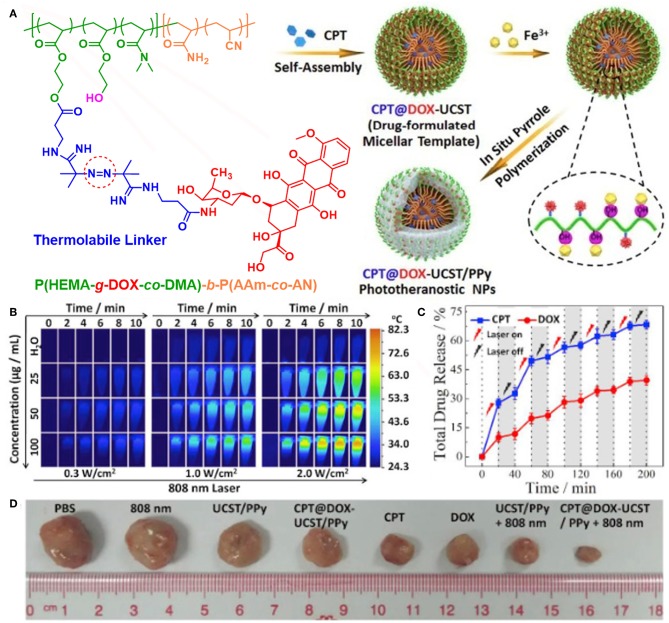
Multifunctional nanoparticles for light-controlled pulsatile drug release in cancer chemo-photothermal therapy. **(A)** Schematic illustration of the synthesis of CPT@DOX-UCST/PPy nanoparticles. **(B)** Thermal images recorded for different contents of CPT@DOX-UCST/PPy upon irradiation with 808 nm laser at different power density. **(C)** *In vitro* CPT and DOX dual drug release profile recorded for CPT@DOX-UCST/PPy with periodic 808 nm laser illumination at 2 W/cm^2^. **(D)** Photographs of tumor tissue of mice upon different treatments. Reproduced with permission from Yang et al. (2018).

In 2012, Liu's group firstly reported poly(vinyl alcohol) (PVA)-stabilized PPy nanoparticles for photothermal therapy (Yang et al., 2012b). Then, they fabricated core-shell structured Fe_3_O_4_@PPy-PEG-DOX nanocomposite by oxidative polymerization of pyrrole on the surface of Fe_3_O_4_ nanoclusters and subsequent surface modification and drug loading (Wang C. et al., 2013). The Fe_3_O_4_ core can be used for MRI to monitor the therapeutic effect, while the PPy shell can generate mild hyperthermia to enhance intracellular delivery of DOX. Remarkable anticancer effect *in vivo* was achieved by combined chemo-photothermal therapy. Other groups also designed various nano-platforms for combined chemo-photothermal therapy by loading drugs in PPy-based nanomaterials, mainly by physical interactions (Wang Y. et al., 2014; Wang J. et al., 2015; Wang K. et al., 2016; Zhu et al., 2016; Chen X. J. et al., 2017; Yao et al., 2017). Very recently, Hu et al. prepared multifunctional PPy/micelle hybrid nanoparticles (denoted as CPT@DOX-UCST/PPy) by polymerizing pyrrole in the shell of polymeric micelles with upper critical solution temperature (UCST) feature (Figure 6; Yang et al., 2018). The polymeric micelles are exquisitely designed by tethering thermo-cleavable DOX prodrug in the corona and encapsulating hydrophobic CPT in the UCST micellar core. CPT@DOX-UCST/PPy had multiple synergistic effects upon NIR light irradiation, which showed great potential to kill three birds with one stone. First, PPy shell can generate heat for photothermal therapy. Second, DOX can be released by cleavage of the thermo-labile linker. Third, CPT can also be released following micellar swelling triggered by phase transition of the UCST core. Together with the photoacoustic (PA) imaging module, CPT@DOX-UCST/PPy can serve as a multifunctional nano-platform for combined chemo-photothermal therapy and theranostics.

Poly(3,4-ethylenedioxythiophene):poly(4-styrenesulfonate) (PEDOT:PSS), commonly used in organic electronics, was developed by Liu's group for photothermal therapy in 2012 (Gong et al., 2013). PEDOT:PSS-PEG nanoparticles were prepared through layer-by-layer coating of charged polymers followed by conjugating with branched PEG. In another work of the same group, chemotherapeutic drugs like DOX and SN38, as well as photodynamic agent Ce6, were encapsulated within PEDOT:PSS-PEG nanoparticles through π-π stacking and hydrophobic interaction. The photothermal effect of PEDOT:PSS-PEG promoted intracellular delivery of DOX, obtaining integrated chemo-photothermal therapy with synergistic effect.

To achieve higher photothermal conversion efficiency, conjugated polymers with narrow energy band gap are highly desired as they are expected to have sharper adsorption peaks to get greater heat generation (He Y. L. et al., 2018). The concept of donor-acceptor (D-A) has been widely applied for designing conjugated polymer-based field-effect transistors and organic photovoltaics. In 2013, Levi-Polyachenko et al. reported for the first time the use of D-A conjugated polymer nanoparticles with low band gap for photothermal ablation of cancer cells *in vitro* (MacNeill et al., 2013). Since then, lots of D-A conjugated polymers have been synthesized for photothermal therapy by proper design of the donor and acceptor structures (Sun et al., 2018). Chemotherapeutic drugs can be co-loaded with hydrophobic D-A conjugated polymers in liposomes or polymeric micelles, or encapsulated in micelles formed with PEG-modified D-A conjugated polymers. These nano-formulations can thus gain significant synergistic effect of combined chemo-photothermal therapy upon NIR light irradiation. For example, Yang's group reported a D-A conjugated polymer PBIBDF-BT with alternating isoindigo derivative bis(2-oxoindolin-3-ylidene)-benzodifuran-dione (BIBDF) and bithiophene (BT) units (Li D. D. et al., 2016). Hydrophobic PBIBDF-BT together with anticancer drug DOX were simultaneously encapsulated in micelles formed by an amphiphilic copolymer poly(ethylene glycol)-block-poly(hexyl ethylene phosphate) (mPEG-b-PHEP). DOX-loaded PBIBDF-BT@NPPPE nanoparticles exhibited NIR-triggered intracellular drug release and synergistic anticancer treatment. Liu and coworkers prepared poly[9,9-bis(4-(2-ethylhexyl)phenyl)fluorene-alt-co-6,7-bis(4-(hexyloxy)phenyl)-4,9-di(thiophen-2-yl)thiadiazolo-quinoxaline] (PFTTQ) with strong absorption in the NIR region (Yuan et al., 2015). An amphiphilic brush copolymer, decorated with 2-diazo-1,2-naphthoquinones (DNQ) moieties and cyclic arginine-glycine-aspartic acid (cRGD), was used to encapsulate hydrophobic PFTTQ and DOX (forming T-PFTTQ/DOX). Upon NIR laser irradiation, the DNQ moieties could undergo hydrophobic-hydrophilic transformation, inducing disassembly of the micelles and subsequent DOX release. At the same time, hyperthermia generated by PFTTQ further contributed to the efficient killing of cancer cells. The combination index was calculated to be 0.48, indicating the synergistic effect of T-PFTTQ/DOX for combined chemo-photothermal therapy. Pu's group synthesized an amphiphilic PEG-grafted poly(cyclopentadithiophene-alt-benzothiadiazole) (PEG-PCB), which could self-assemble into homogenous nanoparticles and simultaneously load anticancer drug DOX via strong hydrophobic and π-π interactions (Jiang et al., 2017). Drug-loaded PEG-PCB (DSPN) could serve as a multifunctional theranosticnanoagent for NIR fluorescence/PA imaging guided chemo-photothermal therapy. Both *in vitro* and *in vivo* results confirmed the superior antitumor efficacy by the synergistic treatment. Similarly, Li et al. prepared several theranosticnanosystems by co-encapsulating diketopyrrolopyrrole-based D-A polymers with anticancer drugs like DOX or curcumin in polymeric micelles or thermosensitive liposomes (Cao et al., 2017a,b). These nanoparticles also exhibited excellent PA imaging-guided chemo-photothermal combined cancer therapy.

### Polydopamine-Based Nanoparticles

Polydopamine (PDA), a melanin-like polymer, can be easily obtained by self-polymerization of dopamine under mild conditions. PDA was introduced by Lee and Messersmith for the first time as a simple and powerful surface functionalization method in 2007 (Lee et al., 2007). Lu's group reported the first application of PDA nanoparticles (PDA-NPs) for *in vivo* photothermal ablation of tumors in 2013 (Liu et al., 2013). PDA-NPs are well-evaluated to have advantages such as strong NIR light absorption, high photothermal conversion efficiency as well as remarkable biocompatibility and biodegradability. Moreover, the surface of PDA-NPs remains high reactive activity for further functional modification. Thiol- and amino-terminated molecules, such as hydrophilic PEG and cancer targeting moieties, can be covalently attached onto PDA nano-surface via Michael addition or Schiff base reactions (Park et al., 2014).

So far, PDA-NPs have been extensively utilized as drug delivery systems for combined chemo-photothermal therapy (Ambekar and Kandasubramanian, 2019; Farokhi et al., 2019). Cheng et al. synthesized PEGylated PDA-NPs (PDA-PEG) to encapsulate anticancer drugs such as DOX and SN38 (Wang X. Y. et al., 2016). Remarkable photothermal effect of PDA-PEG was observed upon 808 nm NIR light irradiation along with enhanced drug release. Drug-loaded PDA-PEG was proved to have synergetic effect on cancer cell killing *in vitro* and tumor suppression *in vivo*. Then, alendronate (ALN)-anchored and SN38-loaded polydopamine nanoparticles (PDA-ALN/SN38) were prepared by the same group, successfully regressing bone tumor and osteolysis by combined chemo-photothermaltherapy (Wang Y. T. et al., 2018). Lu's group prepared PDA nanocomplex (PDA@CP_x_, *x* = 3,6,9) by encapsulating biodegradable coordination polymer (CP) on iron-chelated PDA nanosurface via layer-by-layer method (Chen Y. et al., 2016). DOX loaded PDA@CP_3_ noncomplex was developed for T1/T2 dual mode MRI together with synergistic chemo-photothermal therapy both *in vitro* and *in vivo*, which showed great potential for theranositc nanomedicine.

Benefiting from the excellent surface adhesive capability of PDA, core-shell structured nanocomposites consisting of drug-loaded polymeric core and PDA coated shell were developed for combined chemo-photothermal therapy. Xu et al. reported tumor targeting PLGA/PDA core-shell nanocomposites by coating PDA on DOX loaded PLGA nanoparticles, followed by surface PEGylation and anchoring of Anti-EGFR antibody (He et al., 2017). The PDA shell here not only offered reactive sites for surface decoration but also generated hyperthermia under NIR light irradiation for both photothermal therapy and triggering drug release to improve the synergistic chemotherapy. Nie et al. prepared polymer/PDA nanocomposites by coating PDA on nanoparticles formed from a thermo-sensitive block copolymer P(MEO_2_MA-co-OEGMA-co-DMAEMA)-b-PLGA (Ding et al., 2017). DOX, PTX, and small interfering RNAs were simultaneously loaded within these nanocomposites, generating accurately drug release in response to photothermal effect. These multifunctional nanocomposites integrating photothermal, chemo-, and gene therapy successfully caused regression in triple-negative breast cancer with negligible side effects. Very recently, Dong et al. reported a novel polypeptide nanocomposite PNOC-PDA/DOX by coating PDA on micelles formed by S-nitroso (SNO, a kind of heatsensitive NO donor) conjugated polypeptide copolymer and further loading with DOX (Ding et al., 2019). Upon NIR light irradiation, NO gas was released due to heat-induced S-NO cleavage. The mild hyperthermia together with NO gas therapy were proved to overcome MDR and maximize chemotherapy. The triple chemo-NO-photothermal therapies completely eradicated MCF-7/ADR tumors without skin damage, scarring, and tumor recurrence within 30 days, indicating excellent synergistic effects for reversing MDR in tumors.

**Figure 7:**
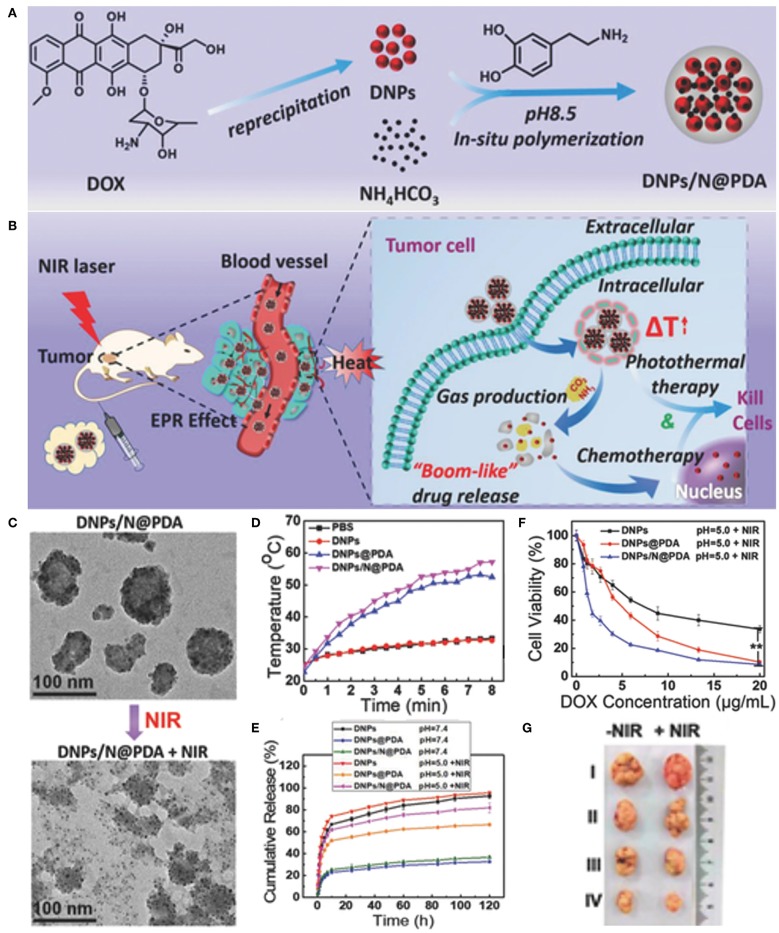
Carrier-free “Nanobomb” for on demand drug release and enhanced chemo-photothermal therapy. **(A)** Schematic illustration of the preparation of DNPs/N@PDA. **(B)** Schematic illustration of the stable blood circulation of DNPs/N@PDA and on demand “bomb-like” drug release and enhanced chemo-photothermal therapy triggered by NIR irradiation. **(C)** TEM images of DNPs/N@PDA before and after NIR laser irradiation (808 nm, 5 W/cm^2^) for 5 min. **(D)** Temperature increase profiles of PBS, DNPs, DNPs@PDA, and DNPs/N@PDA with NIR laser irradiation of 808 nm (5 W/cm^2^, 8 min). **(E)** Cumulative release profiles of DOX from DNPs, DNPs@PDA, and DNPs/N@PDA in PBS with different pHs without or with NIR irradiation (808 nm, 5 W/cm^2^, 5 min). **(F)** *In vitro* cytotoxicity of DNPs, DNPs@PDA, and DNPs/N@PDA at pH 5.0 with NIR laser irradiation (808 nm) of 5 W/cm^2^ for 1 min at different DOX concentrations on HeLa cells after 48 h incubation (**p < vs. DNPs/N@PDA group with NIR irradiation). **(G)** Representative photos of excised tumors 21 d after treatments. Reproduced with permission from Li M. H. et al. (2018).

Carrier-free nanoparticles with high drug loading and on-demand drug release have attracted increasing attention. Liu and coworkers introduced a novel carrier-free “nanobomb” with drug loading efficiency as high as 85.8% (Li M. H. et al., 2018). The “nanobomb” was prepared by two simple steps: the first is making DOX nano-precipitates (DNPs) of ca. 5 nm, and the second is surface deposition of PDA which further induced secondary aggregation of small DNPs to form nanodrugs with an average size of around 70 nm (Figure 7). When exposed to NIR laser, the PDA shell generated enough heat to produce CO_2_ and NH_3_ gases from the encapsulated NH_4_HCO_3_. PDA film outside the DNPs was thus broken up to facilitate *in situ* release of DOX for enhanced chemotherapy. The synergistic photothermal and chemotherapy of the NIR responsive “nanobomb” achieved excellent anticancer activity both *in vitro* and *in vivo*. Recently, Dong's group synthesized high drug-loading PDA-chlorambucil conjugate nanoparticles by direct polymerizing dopamine with a novel pH and reduction-responsive dopamine-chloroambucil prodrug (Du et al., 2019). The PDA-chlorambucil prodrug nanoparticles exhibited triple pH/reduction/NIR light responsive drug release profile *in vitro* and achieved traceless and complete ablation of MCF-7 tumors without recurrence within 50 days by combined chemotherapy and mild hyperthermia.

## Conclusion and Outlook

In this review, we summarized recent advances in NIR light responsive nanomaterials for combined chemo-photothermal cancer therapy. Metal-, carbon-based and organic nanomaterials were included to discuss their design, preparation and application in combined therapy for improving cancer treatment. The combined chemo-photothermal therapy has been widely evaluated to show synergistic anticancer effect in a “1+1>2” manner. On the one hand, hyperthermia induced by photothermal agents upon specific NIR laser irradiation in the tumor region can not only kill cancer cells directly, but also serve as heat trigger to stimulate drug release in a controlled manner and facilitate cell membrane permeability to enhance drug uptake. On the other hand, optimized chemotherapy by systematic administration of anticancer drugs can help to completely eradicate tumors together with photothermal therapy. The combined chemo-photothermal therapy has also shown excellent performance in overcoming MDR and lung metastasis.

In spite of the rapid development and promising potential of nanomaterials for chemo-photothermal therapy, there still exist several critical issues that need to be addressed. Firstly, the photothermal conversion efficiency, which will significantly influence the dosage of photothermal agents and NIR light intensity/irradiation time for efficient heat generation, differs among various nanomaterials concerned in this review. Photothermal conversion efficiencies of several kinds of nanomaterials are summarized in Table 1. More efforts are needed to be put into the creation of novel nanomaterials with favorable photothermal conversion efficiency in order to achieve satisfactory photothermal outcome and reduce the administration dose of nanomaterials. Secondly, though NIR laser can reach deeper tumor tissues, the penetrated depth is still limited. Therefore, the non-invasive photothermal therapy seems mostly feasible for superficial tumors. For internal organ tumors, efficient photothermal therapy can be conducted with minimal intervention by the development of novel invasive medical devices. Thirdly, long-term cytotoxicity of these nanomaterials, especially those with poor biodegradability such as carbon-based nanomaterials, remains uncertain and deserves more attention in future studies. In this regard, organic photothermal agents-based nanomaterials exhibit better performance in biodegradability and biocompatibility. For example, nano-formulations containing FDA-approved ICG are very promising for future clinical utilization. Another concern of cytotoxicity comes from the loaded chemotherapeutic drugs. Though excellent anticancer efficacy can be obtained in a lower dosage with the assistance of hyperthermia, the systematic distribution may still induce undesired side effects. Covalently conjugated prodrugs can significantly decrease premature release of drugs during blood circulation before linker cleavage under tumor physiological environment. Surface tailoring through conjugation of targeting moieties on nanomaterials can further enhance tumor accumulation via specific nano-cell interactions, consequently enhancing anticancer efficacy, and reducing side effects. Lastly, the mechanism of synergistic effect for combined chemo-photothermal therapy needs deeper investigation in the future. Hyperthermia has been reported to increase vascular permeability within tumor tissues, thus promoting drug enrichment and enhancing therapeutic outcome of chemotherapy. Multidrug resistance is one of the major obstacles against efficient cancer treatment by single chemotherapy. Photothermal therapy has been proved to significantly reduce drug efflux by inhibiting P-glycoprotein expression with augmentation of drug sensitivity to cancer cells. Nevertheless, molecular mechanisms beneath chemo-photothermal therapy, involving multiple signaling pathways in cancer cells, are still rarely to be explored. Certainly, studies on tumor biology will help us to have better understanding about intrinsic mechanism for drug resistance, facilitating the design of novel nanomaterials for precise and efficient cancer treatments.

**Table 1:** Photothermal conversion efficiency of various nanomaterials applied in chemo-phototermal therapy.

**Nanomaterials**	**Photothermal conversion efficiency (%)**	**NIR light (nm)**	**References**
Gold nanorods	16.92	808	Manivasagan et al., 2019
Gold Janus nanoparticles	49.5	808	Zhang L. et al., 2016
Cu_2−x_Te nanocubes	25.68	808	Poulose et al., 2016
Cu_2_S nanocrystals	25.3	800	Poulose et al., 2015
MoS_2_/Cu_1.8_S nanosheets	32.5	980	Meng et al., 2017
MoSe_2_ nanosheets	32.8	808	Wang C. et al., 2016
MoSe_2_/Bi_2_Se_3_ nanosheets	59.3	808	Wang Y. et al., 2019
Bamboo charcoal nanoparticles	29.42	808	Dong et al., 2016
Mesoporous carbon nanoparticles	27.4	808	Wang X. et al., 2019
IR780-HSA NPs	10	808	Pei et al., 2019
IR780-CSOSA	33.5	808	Tan et al., 2019
Aza-BODIPY prodrug NPs	38.3	660	Chen et al., 2018
PPy	40	808	Chen X. J. et al., 2017
PEGylated poly-(diketopyrrolopyrrole-thiophene)	76	808	Yao et al., 2017
PBIBDF-BT@NP_PPE_	46.7	808	Li D. D. et al., 2016
PEG grafted poly(cyclopentadithiophene-alt-benz othiadiazole)	30.8	808	Jiang et al., 2017
PDA	40	808	Liu et al., 2013
PDA	33.7	808	Ding et al., 2019

Regardless of the existing challenges, chemo-photothermal combination therapy has shown promising results in many experiments. With the rapid development of nanoscience, material chemistry, and tumor biology, we believe that successful clinical applications of nanomaterials for chemo-photothermal cancer treatment can be expected in the future.

## Author Contributions

LL conceived and coordinated this project. ZL and YC wrote this paper. YYa and YYu collected and summarized literatures. YZhan, DZ, and XY edited pictures in this paper. XO, ZX, and YZhao revised this paper.

### Conflict of Interest

The authors declare that the research was conducted in the absence of any commercial or financial relationships that could be construed as a potential conflict of interest.
